# FOXP3 over-expression inhibits melanoma tumorigenesis via effects on proliferation and apoptosis.

**DOI:** 10.18632/oncotarget.1600

**Published:** 2013-12-20

**Authors:** BeeShin Tan, Matthew Anaka, Siddhartha Deb, Claudia Freyer, Lisa M. Ebert, Anderly C. Chueh, Sheren Al-Obaidi, Andreas Behren, Aparna Jayachandran, Jonathan Cebon, Weisan Chen, John M. Mariadason

**Affiliations:** ^1^ Ludwig Institute for Cancer Research Ltd. Melbourne-Austin Branch, Heidelberg, Victoria, Australia.; ^2^ Department of Pathology, Peter MacCallum Cancer Centre, Melbourne, Victoria, Australia.; ^3^ Centre for Cancer Biology, SA Pathology, Frome Road, Adelaide, Australia.; ^4^ School of Molecular Science, LaTrobe University, Bundoora, Victoria, Australia.

**Keywords:** FOXP3, melanoma, proliferation, apoptosis

## Abstract

The Forkhead box P3 (FOXP3) transcription factor is the key driver of regulatory T cell (Treg cells) differentiation and immunosuppressive function. In addition, FOXP3 has been reported to be expressed in many tumors, including melanoma. However, its role in tumorigenesis is conficting, with both tumor suppressive and tumor promoting functions described. The aim of the current study was to characterize the expression and function of FOXP3 in melanoma. FOXP3 expression was detected by immunohistochemistry (IHC) in 12% (18/146) of stage III and IV melanomas. However expression was confined to fewer than 1% of cells in these tumors. Stable over-expression of FOXP3 in the SK-MEL-28 melanoma cell line reduced cell proliferation and clonogenicity *in vitro*, and reduced xenograft growth *in vivo*. FOXP3 over-expression also increased pigmentation and the rate of apoptosis of SK-MEL-28 cells. Based on its infrequent expression in human melanoma, and its growth inhibitory and pro-apoptotic effect in over-expressing melanoma cells, we conclude that FOXP3 is not likely to be a key tumor suppressor or promoter in melanoma.

## INTRODUCTION

The FOXP3 transcription factor regulates the lineage-specific differentiation of regulatory T cells (Treg cells), a subset of CD4+ T cells crucial for the maintenance of immune homeostasis. FOXP3 expression was originally thought to be restricted to Treg cells [1], however recent studies have suggested that FOXP3 is also expressed in multiple normal tissues, as well as in a number of tumor types. Specifically, FOXP3 expression has been reported in normal breast, prostate and ovarian epithelium, and to be down-regulated in the corresponding tumor tissue [2-4]. Conversely, increased FOXP3 expression has been reported in pancreatic adenocarcinoma [5], melanoma [6-8], hepatocellular carcinoma [9], leukemia [10], bladder cancer [11], thyroid carcinoma [12] and cervical cancer [13], with no expression in corresponding normal tissue. These findings suggest either a pro- or anti-tumorigenic role, depending on the tumor type.

Several additional lines of evidence suggest a tumor suppressor role for FOXP3 in certain tumor types. First, FOXP3 represses expression of the *HER2*, *Skp2*, *SATB1* and *MYC* oncogenes [2, 3, 14, 15], and induces expression of the tumor suppressor genes *p21* and *LATS2* in breast and prostate cancer cells [16, 17]. FOXP3 has also been shown to repress BRCA1-mediated DNA repair and to promote DNA damage-induced apoptosis [18]. Second, over-expression of FOXP3 in glioma, breast, prostate and ovarian cancer cell lines induces profound growth inhibition *in vitro* and *in vivo* [2-4, 19]. Finally, somatic inactivating mutations of *FOXP3* have been reported in breast and prostate cancers [2, 3], although notably these findings were not confirmed by recent whole genome sequencing studies of these tumors [20]. In addition, our group did not identify any mutations in FOXP3 in a panel of 54 early passage melanoma cell lines or well-established breast and prostate cancer cell lines [21].

In contrast, FOXP3 has also been suggested to facilitate tumorigenesis by enabling tumor cells to evade anti-tumor immunity. This has been demonstrated in pancreatic carcinoma and melanoma cell lines, where FOXP3 expression inhibits T cell proliferation in co-culture systems [5, 8]. FOXP3 expression in tumors was also shown to be associated with worse overall survival in breast, bladder, and colorectal cancer patients [11, 22, 23].

We previously demonstrated FOXP3 expression in human melanoma tissue and cell lines [6], although the frequency of its expression was not assessed in a large cohort of cases. In addition, whether FOXP3 promotes or inhibits the growth of melanoma cells is unknown. The objectives of this study were to evaluate the frequency of FOXP3 expression in metastatic melanoma, and to determine its role in regulating the growth and survival of melanoma cells.

## RESULTS

### FOXP3 expression is infrequent in advanced-stage melanoma

**Figure 1:**
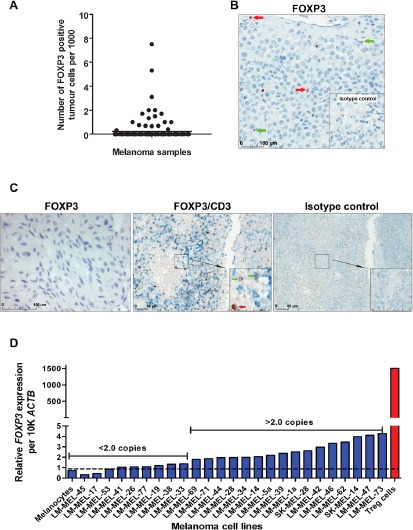
FOXP3 expression in advanced-stage melanoma (A) Distribution of FOXP3 expression in 146 stage III and IV melanomas. (B) Representative image of FOXP3 staining in a melanoma sample using the anti-FOXP3 Ab10563 antibody or IgG isotype control antibody (bottom right panel). Tumor cells with large irregular-shaped nuclei are indicated with red arrows while Treg cells with smaller, denser nuclei are indicated with green arrows. (C) Staining of melanomas and cultured melanocytes with anti-FOXP3 Ab20034 (clone 236A/E7) antibody. Sections were double-stained with anti-FOXP3 (brown) and anti-CD3 (Ferrangi blue) (left panel) or IgG isotype control antibodies (right panel) to distinguish between FOXP3 expressing tumor cells and infiltrating T cells. (D) Detection of FOXP3 mRNA expression in a panel of human melanoma cell lines using quantitative real-time PCR.

We previously reported FOXP3 expression in human melanomas by demonstration of co-staining of FOXP3 with the melanoma cell surface antigen Melan-A [6]. However, this analysis did not quantify the percentage of melanomas which express FOXP3, or the percentage of FOXP3 positive cells within a tumor. To address this, we performed immunohistochemical staining for FOXP3 on a tissue microarray (TMA) comprising tumors from 146 patients with stage III and stage IV metastatic melanoma, using the rabbit polyclonal anti-FOXP3 antibody Ab10563, directed against the C-terminus of FOXP3. Tumor cells and lymphocytes were distinguished based on the morphology of the stained cells (Figure 1A&B). This analysis demonstrated that 18/146 (12%) of advanced-stage melanomas contained FOXP3 positive tumor cells. Quantification of the frequency of FOXP3 positive cells showed this to range between 0.3 and 7.5 positive cells per 1000 tumor cells (0.03-0.75%). To validate these findings using an independent antibody, five of the FOXP3-positive tumors were stained with the mouse monoclonal anti-FOXP3 antibody Ab20034 (clone 236A/E7, directed against amino acids AA107-AA196 of FOXP3). To further distinguish between melanoma cells and Treg cells, sections were co-stained with an anti-CD3 antibody. This analysis confirmed the findings obtained with the rabbit polyclonal antibody, with <1% of melanoma cells staining positive for FOXP3. Finally, we did not observe any FOXP3 staining in normal melanocytes cultured *in vitro* (Figure 1C).

We next examined *FOXP3* mRNA expression in a panel of 25 melanoma cell lines and in normal cultured melanocytes by quantitative PCR (QPCR). Low levels of *FOXP3* mRNA (2-5 copies per 10,000 copies of beta-actin) were detected in 16 of the 25 melanoma cell lines, representing expression 300- to 1000- fold lower than the level observed in Treg cells (2000 copies per 10,000 copies of beta-actin) (Figure 1D). Minimal level of FOXP3 was observed in normal melanocytes.

### Effect of FOXP3 over-expression on melanoma cell growth

**Figure 2:**
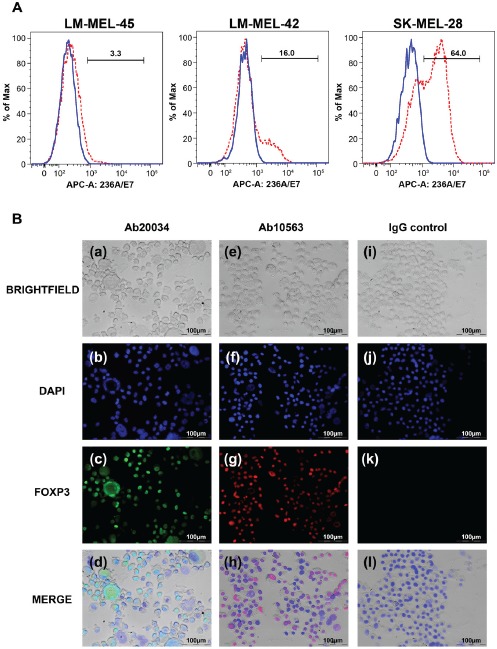
Establishment of FOXP3 over-expressing melanoma cell lines (A) Detection of FOXP3 expression following over-expression in melanoma cell lines by staining with the anti-FOXP3 antibody (clone 236A/E7) and flow cytometry analysis. Blue solid lines represent FOXP3 expression in cells transfected with the pcDNA-empty vector and red dashed lines represent cells transfected with FOXP3. (B) Immunofuorescence staining of FOXP3 nuclear staining in a SK-MEL-28-FOXP3 clone. The anti-FOXP3 antibodies Ab20034 (a,b,c,d) and Ab10563 (e,f,g,h) were used to detect FOXP3 using Alexa-488 (green) and Alexa-555 (red)-conjugated secondary antibodies, respectively. FOXP3 nuclear staining is indicated by the red arrows. Panels i,j,k,h represents the cells stained with an IgG isotype control.

To directly determine the impact of FOXP3 on the growth of melanoma cells, we sought to manipulate FOXP3 levels in melanoma cell lines by RNAi knockdown and vector-mediated over-expression. Reliable down-regulation of FOXP3 in melanoma cell lines proved difficult to accurately evaluate due to its low basal level of expression (data not shown). We therefore sought to generate stable FOXP3 over-expressing melanoma cell lines, and twelve melanoma cell lines were chosen for transfection; LM-MEL-14, LM-MEL-17, LM-MEL-31, LM-MEL-34, LM-MEL-42, LM-MEL-45, LM-MEL-47, LM-MEL-53, LM-MEL-62, LM-MEL-73, SK-MEL-14 and SK-MEL-28. For each cell line, transfection was performed in triplicate. Out of these 12 lines, we were able to establish stable G418 resistant clones from 9 of the lines (LM-MEL-17, LM-MEL-31, LM-MEL-34, LM-MEL-42, LM-MEL-45, LM-MEL-47, LM-MEL-62, SK-MEL-14 and SK-MEL-28). However, eight out of the nine stably transfected melanoma cell lines demonstrated increased FOXP3 expression in <30% of cells (Supplementary Figure 1). SK-MEL-28 was the only cell line for which FOXP3 stable transfectants could be generated in which increased FOXP3 expression occurred in 64% of cells (Figure 2A). Nuclear FOXP3 expression was confirmed in this line by immunofuorescence (Figure 2B).

**Figure 3:**
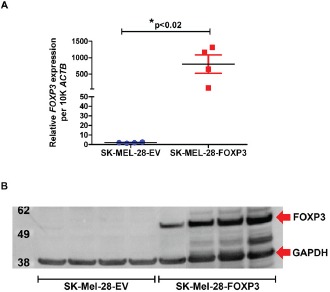
Validation of FOXP3 over-expression in SK-MEL-28 cells (A) FOXP3 mRNA expression in four SK-MEL-28-FOXP3 and SK-MEL-28-EV clones measured using quantitative real-time PCR. (B) FOXP3 protein expression in the same four SK-MEL-28-EV and SK-MEL-28-FOXP3 clones detected by Western Blot using the anti-FOXP3 (236A/E7) antibody. The expected protein size for myc-tagged FOXP3 is 51kDa, and GAPDH is 38kDa.

We next performed single cell cloning of the SK-MEL-28-FOXP3 and SK-MEL-28-empty vector (EV) control lines in an attempt to control for intra-tumoral heterogeneity. Four independent SK-MEL-28-FOXP3 and SK-MEL-28-EV clones were derived for further characterization. *FOXP3* mRNA expression in the SK-MEL-28-FOXP3 clones was 90- to 1100-fold higher than that in SK-MEL-28-EV control clones, with expression levels comparable to that of Treg cells (Figure 3A). FOXP3 protein expression in the SK-MEL-28-FOXP3 clones was also assessed by Western Blot (Figure 3B), which confirmed significantly higher level of FOXP3 protein expression in SK-MEL-28-FOXP3 clones compared to SK-MEL-28-EV clones.

### FOXP3 over-expression increases pigmentation and markers of melanocyte differentiation in SK-MEL-28 melanoma cells

**Figure 4:**
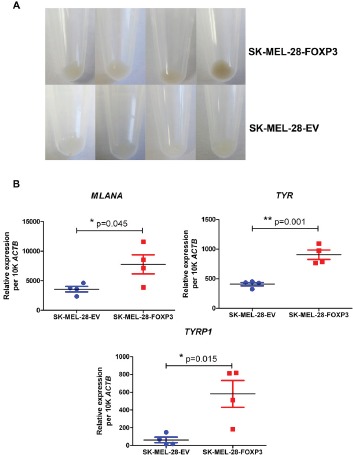
Effect of FOXP3 over-expression in SK-MEL-28 cells on melanoma cell pigmentation (A) Increased pigmentation observed macroscopically in the four SK-MEL-28-FOXP3 clones compared to the SK-MEL-28-EV clones. (B) Expression of the melanoma-specific differentiation genes MLANA, TYR and TYRP1 in the four SK-MEL-28-FOXP3 clones (red) and SK-MEL-28-EV clones (blue). Values shown are the mean and SEM of the four clones

After approximately five passages, we noted that the four SK-MEL-28-FOXP3 clones demonstrated increased pigmentation, whereas none of the four SK-MEL-28-EV clones displayed this feature (Figure 4A). We therefore examined the expression of melanoma-specific differentiation genes including Melan-A (*MLANA*), Tyrosinase (*TYR*) and Tyrosinase-related protein 1 (*TYRP1*) by quantitative PCR. Expression of these genes was significantly up-regulated (~2- to 9-fold) in FOXP3 over-expressing compared to EV clones (Figure 4B).

### FOXP3 over-expression decreases proliferation of SK-MEL-28 melanoma cells

**Figure 5:**
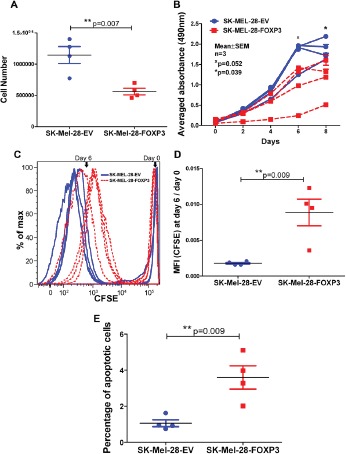
Effect of FOXP3 over-expression in SK-MEL-28 cells on melanoma cell proliferation and apoptosis (A) Cell number. The four SK-MEL-28-EV and SK-MEL-28-FOXP3 clones were seeded at a density of 1x10^5^ cells/T75cm^2^ flask, and cell counts determined five days later. Each data point is the average of three independent experiments of the SK-MEL-28-FOXP3 clones (red) and the SK-MEL-28-EV clones (blue). (B) Assessment of cell proliferation of the four SK-MEL-28-EV and SK-MEL-28-FOXP3 clones using the MTS assay. Values shown are the mean and SEM of a single representative experiment performed in triplicate. (C) Assessment of cell proliferation using the CFSE-Proliferation assay. SK-MEL-28-EV clones (blue dotted lines) showed lower CFSE staining intensity due to a greater number of divisions during the 6 day period compared to SK-MEL-28-FOXP3 clones (red solid lines). (D) Graph represents the mean CFSE intensity (MFI) of the four SK-MEL-28-EV and SK-MEL-28-FOXP3 clones at day 6 relative to day 0 (after labeling). (E) Basal apoptosis was determined in the four SK-MEL-28-EV and SK-MEL-28-FOXP3 clones by propidium iodide staining and flow cytometry.

Three approaches were utilised to compare the proliferative rates of SK-MEL-28-EV and SK-MEL-28-FOXP3 clones; cell counts, MTS assays and CFSE dye dilution experiments. For determination of cell proliferation rates by cell counting, each clone was seeded at the same density and the number of cells counted after five days. Cell number was significantly higher after 5 days in the SK-MEL-28-EV clones compared to the SK-MEL-28-FOXP3 clones (p<0.01; Figure 5A).

MTS colorimetric assays were subsequently performed over an eight day period to verify this finding (Figure 5B). At day 6 and 8 post-seeding, cell viability was significantly higher in the SK-MEL-28-EV clones compared to the FOXP3 over-expressing clones (p≤0.05).

Finally, cells were stained with CFSE and the fluorescence intensity measured after 6 days. The mean intensity of CFSE staining was significantly lower in the four SK-MEL-28-EV clones (blue dots) compared to the SK-MEL-28-FOXP3 clones (red dots), consistent with more frequent cell division in the SK-MEL-28-EV clones (p<0.05) (Figure 5C). Collectively, these findings demonstrate that FOXP3 over-expression slows the growth of melanoma cells.

### FOXP3 over-expression increases apoptosis in SK-MEL-28 melanoma cells

As previous studies in glioma, breast, prostate and ovarian cancers demonstrated that FOXP3 over-expression induces apoptosis [2-4, 19], we examined the effect of FOXP3 over-expression on apoptosis in SK-MEL-28 melanoma cells. SK-MEL-28-FOXP3 and SK-MEL-28-EV clones were stained with propidium iodide and the sub-diploid (apoptotic) fraction assessed by flow cytometry. The basal fraction of apoptotic cells was 3.8fold higher in SK-MEL-28-FOXP3 clones compared to the empty vector controls (Figure 5D).

### FOXP3 over-expression decreases clonogenecity of SK-MEL-28 melanoma cells

**Figure 6:**
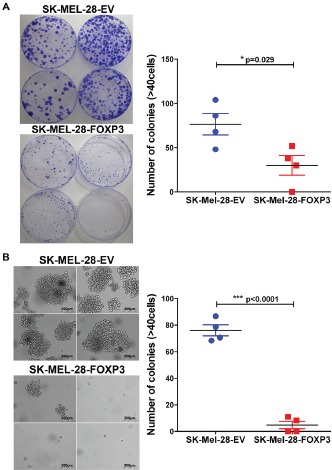
Effects of FOXP3 over-expression on melanoma cell clonogenecity (A) Colony formation of the four SK-MEL-28-FOXP3 and EV control clones grown on plastic. 1,000 cells were seeded in 150mm x 15mm petri dishes and colony formation monitored over 3 weeks. Macroscopic view of the colonies stained with 0.1% Crystal Violet (left panel) and the number of colonies (≥40 cells) counted (right panel). (B) Colony formation of the four SK-MEL-28-FOXP3 and EV control clones grown in soft agar for 3 weeks. Brightfield images of the colonies from four SK-MEL-28-EV and SK-MEL-28-FOXP3 clones (left panel) and the number of viable colonies (≥40 cells) counted after staining with MTT (right panel). Values shown are the mean and SEM of a single representative experiment performed in triplicate.

We next performed clonogenic assays on plastic and in soft agar to test for the ability of FOXP3 over-expressing cells to grow under adherent and anchorage independent conditions, respectively. In adherent colony formation assays, SK-MEL-28-FOXP3 clones demonstrated a marked reduction in both the size and number of colonies compared to SK-MEL-28-EV clones (p<0.05) (Figure 6A). Likewise, the ability of SK-MEL-28-FOXP3 clones to form colonies in soft agar was significantly reduced, with 8-fold fewer colonies observed in the SK-MEL-28-FOXP3 clones (Figure 6B).

### FOXP3 over-expression reduces tumor growth *in vivo*

**Figure 7:**
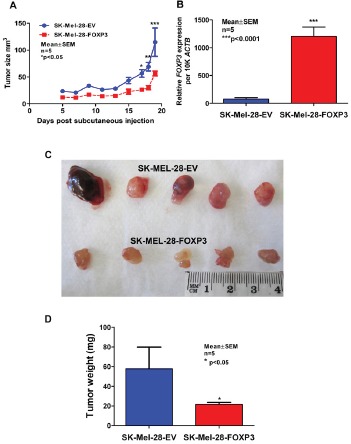
Assessment of growth of SK-MEL-28-EV and SK-MEL-28-FOXP3 clones as xenografts in vivo (A) 9x10^5^ cells from a representative SK-MEL-28-EV and SK-MEL-28-FOXP3 clone were injected into BALB/c nude mice and tumor growth monitored daily by calliper measurements (n=5). (B) Validation of FOXP3 mRNA expression in the resected tumors by quantitative real-time PCR (QPCR) (right panel). (C) Images of resected tumors and (D) weight of the SK-MEL-28-FOXP3 and SK-MEL-28-EV tumors measured following resection after sacrifice. The values shown are the mean and SEM of the five tumors.

To validate the *in vitro* finding that FOXP3 over-expression slows the growth of melanoma cells, a representative SK-MEL-28-FOXP3 clone with high FOXP3 expression and a representative SK-MEL-28-EV clone were selected for assessment of growth *in vivo*. Equal numbers of cells from both clones were injected subcutaneously into Balb/c nude mice and tumors allowed to develop for 3 weeks. The growth rate of the SK-MEL-28-FOXP3 clone was significantly slower than that of the empty vector clone (Figure 7A). Consistent with this finding, resected SK-MEL-28-FOXP3 tumors had a significantly lower weight compared to SK-MEL-28-EV control tumors (Figure 7B). FOXP3 expression in the tumors was determined by QPCR, which confirmed that the SK-MEL-28-FOXP3 clone retained expression of FOXP3 during this assay.

### Microarray expression profling of FOXP3 over-expressing SK-MEL-28 melanoma cells

**Figure 8:**
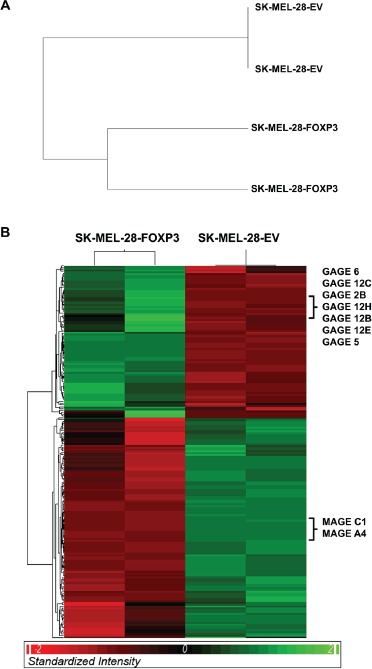
Gene expression profiling of SK-MEL-28-FOXP3 and SK-MEL-28-EV clones (A) Hierarchical clustering of the entire unfiltered gene expression dataset effectively separated the SK-MEL-28-FOXP3 and SK-MEL-28-EV clones. (B) Hierarchical clustering of the 204 significantly differentially expressed genes.

Lastly, to gain mechanistic insights into the molecular basis for FOXP3-mediated growth inhibition and apoptosis, we used Illumina HT-12 gene expression microarrays to interrogate the transcriptomes of two SK-MEL-28-FOXP3 and two SK-MEL-28-EV clones. FOXP3 over-expression led to a clear and consistent difference in the gene expression profile, as demonstrated by separation of the SK-MEL-28-FOXP3 and SK-MEL-28-EV clones by hierarchical clustering (Figure 8A). We identified 204 differentially expressed genes between the two groups when the percentage of false positives was limited to 5% (Supplementary Table 2) (Figure 8B). Twelve of the differentially expressed genes were validated by QPCR, and all demonstrated the same differential expression pattern as that observed in the microarray (Supplementary Figure 2). FOXP3 was not found among the differentially expressed genes, as the FOXP3 probes on the HT-12 array target the FOXP3 3' UTR which is not present in the expression vector. At the level of individual genes, one of the most highly up-regulated was MT1F (Metallothionein 1F), a gene involved in cellular responses to a variety of stressors [24], suggesting that FOXP3 over-expression may be inducing cellular stress in these cells.

Notably, some of the differentially expressed genes with the greatest fold changes were members of the Cancer Testis Antigen gene families, which are known to be epigenetically regulated and aberrantly re-expressed in melanoma and other cancers [25]. In particular several *GAGE* family members were highly up-regulated, and several MAGE family members such as *MAGEC1* and *MAGEA4* were highly down-regulated. Despite the differences in cell proliferation and apoptosis, no differential expression of typical regulators of proliferation and apoptosis was observed.

## DISCUSSION

FOXP3 expression has been reported in a variety of normal and cancerous tissues outside of the Treg cell lineage, with two opposing expression patterns. On the one hand, FOXP3 expression has been reported to be widespread in the normal epithelium and tissue of the breast, prostate, ovary and brain, and down-regulated in matched tumor cells [2, 4, 19, 26], although in mice, the level of *Foxp3* mRNA expression in these tissues was shown to be approximately 100-fold lower than that in Treg cells [27]. Conversely, FOXP3 has been reported to be over-expressed in tumor cells in pancreatic adenocarcinoma, melanoma, leukemia, hepatocellular carcinoma, bladder cancer, thyroid carcinoma and cervical cancer [5, 6, 9, 11-13, 28].

In our previous study, we reported FOXP3 expression in both metastatic melanoma tissue samples and cell lines derived from tumor samples [6]. Two other groups subsequently reported similar findings [7, 8]. Niu et al. [8] reported that FOXP3-expressing melanoma cells inhibited the proliferation of anti-CD3/anti-CD28-activated T-cells through FOXP3-induced expression of T cell inhibitory molecules on melanoma cells (B7-H1 and Fas ligand) and secreted immunosuppressive factors (TGF-β). Quaglino et al. [7] demonstrated a significant association between FOXP3 expression in primary melanomas and development of visceral metastases. However, none of these studies systematically addressed the percentage of melanomas which express FOXP3, or the percentage of FOXP3+ cells within melanomas.

We found that 12% of stage III and IV metastatic melanomas expressed FOXP3 based on IHC staining of a tissue microarray. Furthermore, only a small population of cells within these tumors (<1%) expressed FOXP3 with nuclear localization. These results are in keeping with our previous study, where 6-colour flow-cytometry was used to analyse FOXP3 expression within disaggregated metastatic melanoma tissue. In this analysis, a distinct population of melanoma cells (identified as MCSP+ CD3-CD4- CD31- CD90-) expressing high levels of FOXP3 was identified in some samples, with the frequency of these FOXP3^bright^ cells ranging from 0 – 0.24% [6]. Interestingly, for most samples, we were also able to detect low-level FOXP3 expression in the bulk melanoma cell population, detected as a shift in fluorescence intensity compared to the fluorescence-minus-one (FMO) control. We hypothesise that the rare FOXP3^bright^ cells in our previous study correspond to the rare FOXP3^+^ cells identified by IHC in the present study, whereas the low level FOXP3 expression observe in the bulk population by flow cytometry is below the detection limit of IHC. This low level expression is maintained by melanoma cell lines and can be detected at both the gene and protein level. However, we show here that this expression level is several orders of magnitude less than that observed in Treg cells.

The prevalence of FOXP3 staining within melanoma tissue sections in our IHC study is lower than that reported by Niu et al. [8] and Quaglino et al. [7], who reported abundant FOXP3 expression in both the nucleus and cytoplasm of melanoma tissue sections. An important difference in our studies is the FOXP3 antibodies used. While we used the anti-FOXP3 Ab20034 and Ab10563 antibodies, the Niu and Quaglino studies used the anti-FOXP3 PCH101 antibody [7, 8]. A number of recent publications have highlighted the importance of the antibody used in examining FOXP3 protein expression. For example, Tran et al. and Woo et al. reported that commercially available antibodies targeting different FOXP3 epitopes resulted in different staining patterns [29, 30]. The study by Tran et al. [29] also concluded that PCH101 was a less reliable indicator of FOXP3 expression in human activated CD4+ T cells. In the study by Woo et al. [30], the anti-FOXP3 Ab20034 (clone 236A/ E7) antibody which was used in the current study was compared with the anti-FOXP3 Ab22510 antibody, and the authors concluded that more specific staining was obtained with Ab20034 in cervical intraepithelial neoplasia tissue sections. Finally, while FOXP3 expression has been reported in normal thymus as well as lung, ovarian, breast and prostate epithelium [2-4, 27], other studies have demonstrated otherwise, and attributed the differences to immunohistochemical staining artifacts [31-35]. While we did not directly compare the variation in staining of different FOXP3 antibodies in this study, these prior studies indicate that the 236A/E7 clone (Ab20034) is the most suitable antibody for IHC detection of human FOXP3 in both tumor and Treg cells. Based on our current findings generated using anti-FOXP3 antibodies Ab20034 and Ab10563, we conclude that FOXP3 is expressed in 12% of human metastatic melanomas with expression restricted to <1% of cells within these tumors.

Given the low basal expression of FOXP3 in melanoma cell lines, our only means of assessing FOXP3 function in these cells was to over-express the gene. Over-expression of FOXP3 in the SK-MEL-28 cell line increased the level of pigmentation and up-regulated expression of genes involved in pigment production (*MLANA*, *TYR* and *TYRP1*). Notably, these genes are targets of the micropthalmia-associated transcription factor (MITF), a key driver of melanocyte differentiation [36]. Expression of MITF however, was not significantly up-regulated following FOXP3 over-expression (data not shown). MITF has been shown to undergo post-translational modifications that regulate its transcriptional activity [37-39]. A possibility therefore is that FOXP3 over-expression leads to post-transcriptional modification of MITF and subsequent induction of MITF target genes.

We also observed that FOXP3 over-expression markedly reduced the proliferation of SK-MEL-28 cells *in vitro*, reduced their ability to form colonies on plastic and in soft agar, and to grow as xenografts *in vivo*. These anti-tumor effects of FOXP3 in melanoma cells are consistent with that previously reported in gliomas, breast, prostate, and ovarian cancer cells [2-4, 19]. FOXP3 has been shown to exert its growth inhibitory effect on breast and prostate cancer cells by transcriptionally repressing the expression of specific oncogenes (*HER2*, *SKP2*, *SATB1* and *MYC*) [2, 3, 14, 15], and inducing the expression of specific tumor suppressor genes (*CDKN1A*, *LATS2*) [16, 17]. In our gene expression profiling experiments, we did not identify similar regulation of melanoma oncogenes and tumor suppressors or classical regulators of cell cycle and cellular senescence. Thus the mechanism by which FOXP3 inhibits growth of melanoma cells appears distinct.

SK-MEL-28-FOXP3 over-expressing clones also exhibited a higher basal rate of apoptosis. The specific mechanistic basis for this increased sensitivity is unknown, but could refect the induction of cellular stress following FOXP3 over-expression. Re-expression of a gene out of context, for example in a cell lineage where it is not normally found, could disrupt normal homeostatic regulatory processes. This is particularly plausible in the case of transcription factors, where over-expression could change the expression of myriad other molecules, all of which could potentially perturb cellular function. Therefore, it is possible that over-expression of FOXP3 and the subsequent modification of gene expression perturbed normal cell functions in melanoma cells, leading to induction of stress response pathways. For example, sustained stress of the endoplasmic reticulum and cytochrome c release due to over-production of random proteins results in collapse of mitochondrial membrane potential [40]. This possibility is supported by the observation that the classical stress-response gene *MT1F* is up-regulated in FOXP3 over-expressing cells.

The results of the present study, together with our previous report [6] reveal that FOXP3 is expressed at a high level in only a small fraction of melanomas, and in only a minor fraction of cells (<1%) within this subset of tumors. On the other hand, low level FOXP3 expression (likely below the limit of detection by IHC) is observable by flow cytometry and PCR in most freshly isolated melanoma specimens and in most melanoma cell lines. We propose that low level FOXP3 expression is advantageous to the tumor, possibly by providing immune suppressive function, in keeping with previous studies [5, 8, 11, 13, 22]. In contrast, the results shown here clearly demonstrate that FOXP3 over-expression suppressed proliferation, increased differentiation and apoptosis and reduced tumorigenesis *in vivo*. Thus, tumor cells must carefully fine-tune their level of FOXP3 expression to balance these negative effects on growth against the putative positive effects of low level expression on immune escape, potentially explaining the conflicting reports regarding FOXP3 function in cancers. We propose that perturbing this strict regulation represents an exciting therapeutic opportunity.

## MATERIALS AND METHODS

### Cell culture

The LM-MEL-# melanoma cell lines [41] were generated from surgically excised melanoma as described previously [42]. Tissue donors provided informed consent for use of their tumor tissue in these studies and all protocols were approved by the Austin Health Human Research Ethics Committee, Melbourne Australia. Cell lines were maintained in complete RF10 medium which comprised RPMI-1640 supplemented with 2 mM Glutamax, 10mM HEPES, 100 units/mL penicillin/ strepto*MYC*in, (all from Life Technologies; Carlsbad, California, USA) and 10% fetal bovine serum (FBS) (Thermo Fisher Scientific; Waltham, Massachusetts, USA). Normal epidermal melanocyte cultures were obtained from Lonza Biosciences (Basel, Switzerland) and cultured according to the manufacturer's recommendation.

### FOXP3 over-expression construct and transfection

The plasmid encoding wild-type *MYC*-tagged FOXP3 (pENTR-FOXP3) [43] was obtained from Addgene, and the *FOXP3* gene subcloned into pcDNA3.2/ V5-DEST using the LR clonase enzyme of the Life Technologies Gateway Cloning System. Cells were transfected using Lipofectamine 2000 (Life Technologies). Stably transfected cells were selected for and maintained in medium containing 0.4mg/ml – 1.0mg/ml G418, with colonies isolated using cloning cylinders (Merck; Whitehouse Station, New Jersey, USA). Knockdown of FOXP3 expression was performed using multiple siRNAs, the details of which are provided in Supplementary Information.

### Immunofluorescence

Cells were spun onto glass slides using a Cytospin centrifuge (Shandon; Runcorn, UK), then air-dried, fixed with methanol at -20°C for 10 minutes, and washed with ice-cold PBST (PBS with 0.05% Tween-20). 5% bovine serum albumin (BSA) (Sigma; St Louis, Missouri, USA) was used for blocking. Staining with anti-FOXP3 antibody (Ab20034; 2.5µg/ml and Ab10563; 0.8µg/ ml – both Abcam) was followed by incubation with Alexafluor (488 or 555)-conjugated secondary antibodies (Life Technologies). Slides were stained with DAPI (1:50, Sigma; St Louis, Missouri, USA) for nuclear visualization and coverslips added using Vectashield (Vector Laboratories; Burlingame, California, USA).

### Immunohistochemistry

Immunohistochemistry was performed on tissue microarrays constructed from 146 formalin-fixed paraffn-embedded (FFPE) stage III and IV melanomas after EDTA or citrate buffer (pH 8.0) retrieval using the Dako Envision+ kit (Dako; Coopenhagen, Denmark). Sections were incubated with 1X protein blocking buffer for 10 minutes or one hour at room temperature (Dako), followed by primary antibody incubation (Ab20034; 2.5µg/ml; overnight; and Ab10563; 0.8µg/ml) for one hour at room temperature. DAB (3, 3'-diaminobenzidine) was used as chromagen (Vector Laboratories), slides were counterstained with hematoxylin and scanned using a ScanScope XT (Aperio).

### Intracellular FOXP3 staining and flow cytometric analysis

Staining was performed using anti-FOXP3 antibody clone 236A/E7 and the FOXP3 buffer set (eBioscience; San Diego, California, USA) following staining with Live/Dead® Fixable dead cell stain (Life Technologies) at 4°C for 20 minutes. Non-specific staining was reduced by incubation with normal mouse serum (Sigma). Stained cells were acquired on a FACS Canto II instrument (BD Biosciences; San Jose, California, USA), and the data analyzed using the FlowJo software (Tree Star Inc; Oregon, USA).

### Proliferation assays

Cell counts, MTS assay and CFSE decay experiments were performed using standard protocols. Details are provided as Supplementary Information.

### Clonogenecity assays

Adherent colony formation was assessed by low density plating in petri dishes and anchorage independent growth assessed using soft agar assays. Details are provided as Supplementary Information.

### Xenografts

Animal experiments were approved by the Austin Health Animal Ethics Committee. Balb/C nude mice, 4-5 weeks of age were purchased from the Animal Resources Centre (ARC, Perth). 9x10^5^ cells were injected subcutaneously into the right flank of each mouse in 100µl of a 1:1 mixture of RF10 with 1.0mg/ml G418 and matrigel (BD Biosciences, San Jose, California, USA). Tumor size was measured daily and tumor volume was calculated as: tumor volume = ½ (width^2^ x length) [44]. The experiment ended when the first tumor in the cohort reached the pre-determined endpoint of 1cm^3^.

### Cell cycle analysis

Sub-confuent cells were harvested, washed in cold PBS, and resuspended in 500µl propidium iodide solution consisting of 50µg/ml of propidium iodide, 0.1% sodium citrate, 0.1mg/ml RNAse A and 0.05% of Triton-X (all Sigma) in PBS. Cells were incubated overnight at 4°C and analyzed by flow cytometry using the FL2 channel. FlowJo cell cycle histogram analysis was used to quantify the apoptotic cell population.

### Quantitative reverse transcriptase-PCR (QPCR)

Total RNA was extracted using the RNeasy mini kit (Qiagen, Hilden Germany), and reverse transcribed into cDNA using the High Capacity cDNA Reverse Transcription Kit (Life Technologies, Carlsbad, California, USA). QPCR was performed using the SensiFAST SYBR Lo-ROX kit (Bioline, London, UK), and a Stratagene Mx3005P thermocycler. Primer sequences are provided in Supplementary Table 1.

### Gene Expression Microarrays and Data Analysis

Purified RNA was sent to the Australian Genome Research Facility for hybridisation to Illumina HT-12 gene expression microarrays. Details of the data analysis are provided in the Supplementary Information.

### Western Blot

Cells were lysed in RIPA buffer (Sigma) and protein concentrations quantified using the BCA protein assay (Thermo Scientific). Samples were separated using NuPAGE 4-10% BisTris gels (Life Technologies) and MES [2-(N-morpholino) ethanesulfonic acid] SDS running buffer (Life Technologies). Proteins were transfered to PVDF membrane (Milipore) used a semi-dry transferblot (Bio-Rad Laboratories, Hercules, California, USA). Blocking was performed with 5% skim milk, followed by incubation with anti-FOXP3 antibody (236A/ E7 at 2µg/ml from e-Bioscience or anti-GAPDH 1:1000, from Sigma) overnight at 4°C. Horseradish peroxidase-conjugated secondary antibodies (anti-mouse from Sigma St Louis, Missouri, USA or anti-rabbit from New England, Boston, Massachusetts, USA) were then added for one hour at room temperature. Signal detection was performed by incubation of ECL Plus reagent (Amersham BioSciences, Amersham, UK) for 5 minutes at room temperature followed by imaging using the STORM 840 v2005 (Amersham).

### Statistical analysis

All statistical comparisons were performed using a Student's two-tailed t-test using Prism software (GraphPad Software Inc, San Diego, California, USA). A p-value of <0.05 was considered statistically significant.

## Supplementary Figures and Tables




