# Supplementary material for: FOXP3 over-expression inhibits melanoma tumorigenesis via effects on proliferation and apoptosis
Source: Oncotarget. 2013 Dec 20;5(1):264–76. doi: 10.18632/oncotarget.1600 (PMC3960207; doi:10.18632/oncotarget.1600)
Supplement: Supplementary file 1 [file oncotarget-05-0264-s001.pdf]

FOXP3 over-expression inhibits melanoma tumorigenesis via effects on proliferation and apoptosis – Tan et al

Supplementary Figures

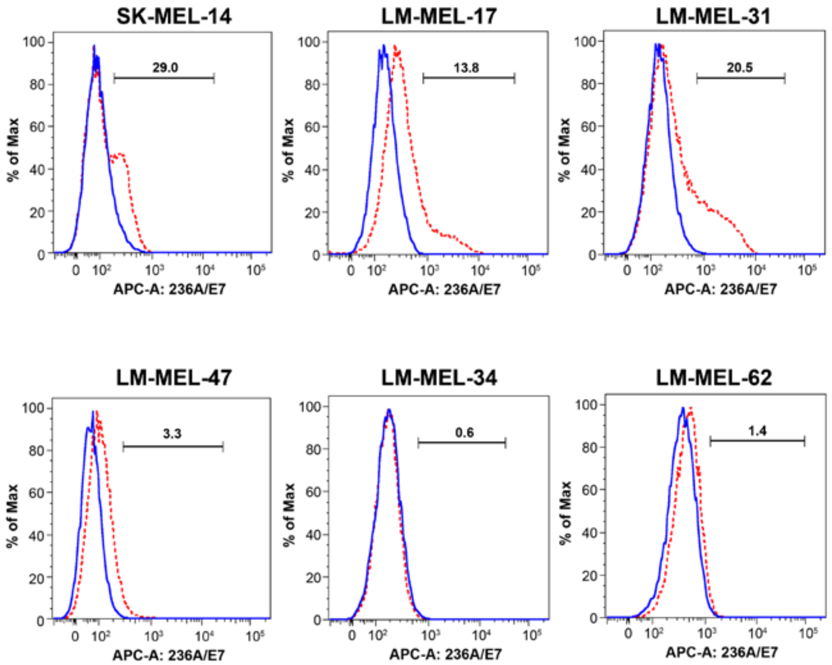

Supplementary Figure 1: Assessment of FOXP3 expression following stable over-expression in the other six melanoma cell lines by flow cytometry. Blue solid lines represent FOXP3 expression in cells transfected with empty vector and red dashed lines represent FOXP3 expression in cells transfected with FOXP3.

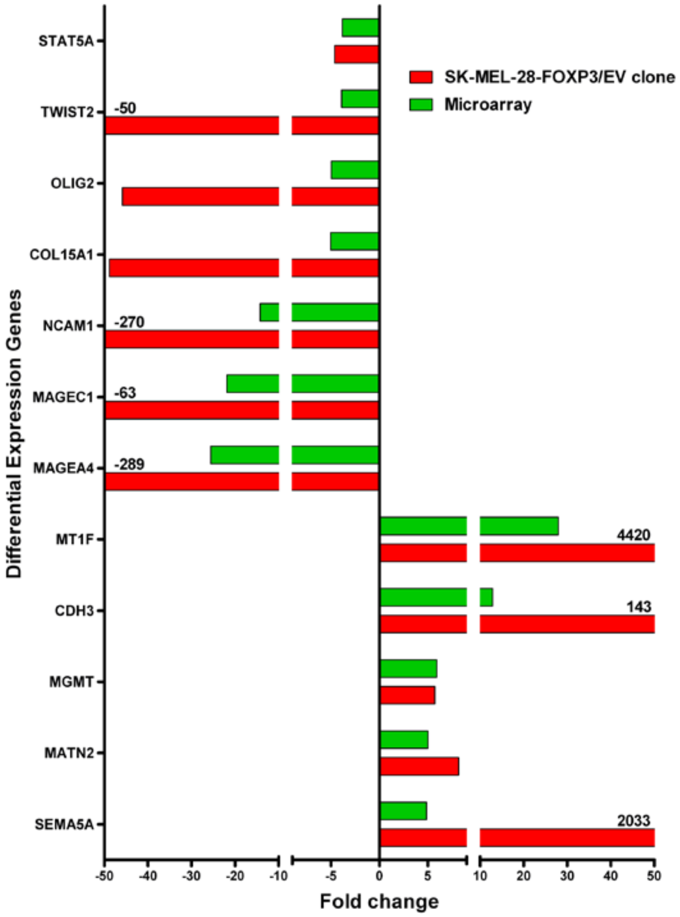

Supplementary Figure 2: QPCR validation of 12 genes identified as differentially expressed between SK-MEL-28-FOXP3 and SK-MEL-28-EV clones by microarray analysis. Shown are the fold change values obtained by microarray (green bars) or QPCR (red bars) from the SK-MEL-28-FOXP3 clone with the highest FOXP3 expression compared to a representative SK-MEL-28-EV clone.

Supplementary Methods

RNAi Knockdown

Small interfering RNAs (siRNAs) targeting *FOXP3* and non-targeting control siRNAs were obtained from Integrated DNA Technologies (IDT) (Coralville, Lowa, USA), Dharmacon FOXP3 siRNA SMARTpool #L-009307-00-0005 (Waltham, Massachusetts, USA) and Ambion FOXP3 siRNA SMARTpool # 4331182 (Life Technologies). The FOXP3-targeting siRNAs described by *Hinz et al.* were synthesized by IDT. Lipofectamine RNAiMAX (Life Technologies) was used as a transfection agent, and the final siRNA concentration was 30nM.

Proliferation Assays

Cell counts: Cells were seeded at a density of $1x10^5$ cells per $T75cm^2$ flask and live cell counts were performed on day 5 using Trypan blue exclusion.

MTS Assay: Cells were seeded at $1x10^3$ cells per well in 24-well plates and the quantity of viable cells measured using the MTS-colorimetric CellTiter 96 Aqueous One Solution (Promega, Madison, USA) every two days. Absorbance was measured at 490nm using a VersaMax absorbance reader and SoftMax Pro software (both from VersaMax, Sunnyvale, California, USA).

CFSE Decay: Cells were washed once in pre-warmed Carboxyfluorescein succinimidyl ester (CFSE) buffer (0.1% FBS in PBS) and resuspended in CFSE buffer. CFSE (Life Technologies) was used at a final concentration of 10µM. Cells were incubated for 10 minutes at 37°C, then washed twice with cold RF10 medium. Fluorescence intensity was measured using the FACS Canto II as above.

Clonogenecity Assays

Adherent colony formation was measured by seeding $1x10^3$ cells/5ml G418-selection media in 10 cm petri dishes. When colony formation was clearly visible in one of the conditions, all plates were fixed in 4% paraformaldehyde and stained with 0.1% Crystal Violet (Sigma). Quantification was performed using TotalLab Quant software (TotalLab, Irvine, California, USA).

Anchorage independent growth was measured using soft agar assays. Briefly, $1x10^3$ cells were mixed in 500µl of 0.9% agar pre-diluted 1:1 with 2x RF10, layered on to a 1.4% agarose base layer diluted 1:1 with 2x RF10. G418-containing RF10 was placed on top of the agarose layers and changed every 3-4 days, with colonies allowed to form for up to 3 weeks. Colonies were stained using MTT (3-(4,5-Dimethylthiazol-2-yl)-2,5-diphenyltetrazolium bromide) for visualization. Colonies comprising ≥40 cells were quantified.

Microarray Data Analysis

Raw data were read into R environment for statistical computing (http://www.r-project.org) and pre-processed using the Bioconductor package Limma [45]. Background correction was performed using the *normexp* function [46], and data were then log-transformed and quantile normalized. Differential expression was determined in these experiments using the rank products method [47]. Clustering and principal component analyses were performed using the Partek Genomics Suite.
